# China's NMPA perspective on the clinical performance of SARS-CoV-2 antigen test reagents

**DOI:** 10.4155/bio-2021-0176

**Published:** 2022-02-21

**Authors:** Yunfeng Lv, Yu Gao, Jingyun He, Chao Xu, Rongzhi Liu, Shengwei Zheng, Li Fang, Congyin Han, Ran Li

**Affiliations:** ^1^Center for Medical Device Evaluation, NMPA, Beijing, 100081, PR China

**Keywords:** Ag-RDTs, antigen test, clinical evaluation, COVID-19, SARS-CoV-2

## Abstract

The COVID-19 pandemic continues to spread all over the world. In the process of emergency use authorization, the Center for Medical Device Evaluation of the China National Medical Products Administration issued ‘Key Points of Technical Review for the Registration of SARS-CoV-2 Antigen/Antibody Detection Reagents’ as the guidance of registration of antigen and antibody test reagents for the industry. In this document, clinical evaluation requirements of antigen detection reagents are elaborated. Based on the Key Points document and the authors’ review practice, this article explains the evaluation methods and requirements of clinical performance of SARS-CoV-2 antigen-detecting rapid diagnostic tests, then analyzes the application scenarios and intended use of antigen detection reagents.

As the COVID-19 pandemic continues to spread all over the world [1,2], many jurisdictions have urgently authorized or approved severe acute respiratory syndrome coronavirus 2 (SARS-CoV-2) diagnostic reagents. In the global diagnostic standards for COVID-19, nucleic acid amplification tests (NAATs) are used as the most important detection method. However, many countries have approved or urgently authorized some antigen detection reagents [3,4]. As a traditional pathogen detection method, antigen detection reagents are immunological methods based on antigen–antibody reactions. The methodologies currently used mainly include chemiluminescence and immunochromatography, among others [5]. The sensitivity of antigen detection is generally lower than that of NAATs, and there are certain differences between the requirements for sample collection and storage in antigen detection and NAATs [6]. However, both antigen detection and NAATs are methods that target the virus itself, so they can be used as direct evidence of infection.

Antigen-detecting rapid diagnostic tests (Ag-RDTs) using the immunochromatographic method are the most widely used antigen detection reagents [7]. NAATs have higher sensitivity and specificity; but they also have higher requirements for laboratory and equipment setup, with more complex operation. Ag-RDTs have lower detection sensitivity; however, they have many advantages, such as easy storage and transportation, simple operation, and low requirements for equipment and the environment. Because of these characteristics of Ag-RDTs, many countries have approved SARS-CoV-2 Ag-RDTs on the market [8,9].

The characteristics of Ag-RDTs require some unique considerations in the premarket clinical performance evaluation of these products. The Center for Medical Device Evaluation of the National Medical Products Administration (NMPA) issued ‘Key Points of Technical Review for Registration of SARS-CoV-2 Antigen/Antibody Detection Reagents’ [10], which, as a guidance of registration, provides a detailed description of the premarket evaluation methods and indicators for evaluation for such products [11]. Previously, the authors published an article that analyzed the requirement for antibody detection reagents [12]. Based on the Key Points document and the authors’ review practice, this article focuses on the clinical research of antigen detection reagents to explain the evaluation methods and requirements of clinical performance of SARS-CoV-2 antigen detection reagents, then analyzes the application scenarios and intended use of antigen detection reagents in the diagnosis of SARS-CoV-2 infections.

## Key points of clinical research on SARS-CoV-2 antigen test reagents

The purpose of clinical performance studies of *in vitro* diagnostic reagents is to confirm the intended use of the product stated in the labeling. For SARS-CoV-2 antigen detection reagents [13], the purpose of clinical performance research is to confirm the consistency of the test results with the infection status of the subjects. Therefore, it is important to focus on the determination of clinical reference standard, subject enrollment criteria, sample collection, sample size determination, completeness of clinical trial data and statistical analysis requirements to ensure that the clinical performance results obtained from clinical research can adequately reflect the performance of the product in the clinical use scenario after the product is launched on the market. Main considerations in the design of such premarket clinical trials are described in detail in the Key Points document.

### Determination of clinical reference methods

The purpose of *in vitro* diagnostic clinical trials is to evaluate the correlation between the test results of the reagents and the disease or condition to be diagnosed. For antigen detection reagents, it is necessary to evaluate the sensitivity and specificity of the antigen test in subjects with suspected COVID-19. Antigen test results should first be compared with the results of clinical diagnostic criteria. During clinical testing, the clinical diagnostic criteria should be the basis of the reference system in the diagnosis and treatment plan (guidelines) for the COVID-19. Consequently, the Key Points document stipulates the diagnosis and treatment guidance issued by the National Health Commission of China as the reference system [14], including definitions of suspected cases and clinical diagnostic criteria. Criteria for diagnosis include computed tomography imaging, epidemiological history, clinical symptoms and laboratory testing results.

Although SARS-CoV-2 antigen reagents are used to detect pathogen infection, there are differences in sensitivity and other performance of immunological methods compared with commonly used NAATs. Analysis of the performance gap between antigen detection results and nucleic acid test results is important in understanding the role of antigen detection in the diagnosis of COVID-19. Therefore, in the clinical trial of antigen detection reagents, the nucleic acid test results of the samples should be collected simultaneously in addition to comparison experiments with clinical diagnostic reference standards.

### Subject requirements

The enrolled subjects of the clinical trial should be able to represent the intended population for the reagent. In clinical trials of Ag-RDTs, suspected cases of COVID-19 should be included, and the applicable scenarios of antigen detection should be fully considered. The application scenario of antigen detection is generally to find early infections faster in clinical or epidemic prevention inspections rather than evaluation and monitoring in the treatment process of clinical institutions. In remote and backward areas where there are no PCR instruments, antigen detection is a good choice. Therefore, subject selection should be based on the characteristics of the clinical application scenarios with a focus on the initial infection cases rather than the treated cases. The initial stage of infection is patients with clinical symptoms within 1 week. Suspected cases are identified as either ‘confirmed cases’ or ‘excluded cases’ based on the clinical diagnostic criteria in the diagnosis and treatment guidance issued by the National Health Commission of China.

### Sample collection requirements

Ag-RDTs are expected to use throat swab and nasopharyngeal swab samples. Freshly collected samples should be used. Samples should be collected, preserved and transported in accordance with the requirements of the clinical trial protocol using reagents and methods that have been validated for Ag-RDTs. The antigen detection and synchronous NAATs should be carried out in a timely manner.

### Sample size

It is recommended that the size of the enrolled samples be estimated using an appropriate statistical model considering the preset values of clinical evaluation indicators such as clinical sensitivity and specificity, as well as related statistical parameters. The presets for the clinical evaluation indicators should be verified based on laboratory research and clinical requirements. Based on the preceding clinical evaluation indicators, at least 200 patients diagnosed with COVID-19 at the initial stage of infection and 300 suspected patients eventually ruled out of infection should be included. It should be noted that the inclusion of positive patients should be as much as possible to cover the different times of the initial infection. It should be avoided to include patients at a certain time point, such as 3–5 days after infection, and to avoid centralized inclusion of strong positive samples. This will help minimize the selection bias and reduce systematic errors.

### Data integrity requirements

Clinical trial data should include detailed and complete clinical background of the subject, including the time of onset of clinical symptoms, time of visit, time when samples were taken, sample type, and time of the antigen and nucleic acid testing as well as results. All of the information is important.

### Statistical analysis

The purpose of clinical trials on these products is to verify whether declared product results are consistent with clinical findings and to evaluate whether product performance meets clinical needs. In general, by comparing the confirmed and excluded results of the COVID-19 testing, 2 × 2 contingency tables in diagnostic tests are used to display the results and calculate clinical sensitivity and clinical specificity, and confidence intervals are used for statistical analysis of clinical trial data.

To evaluate the clinical performance of antigen reagents in clinical application scenarios, the antigen test results are compared with the NAAT results of the same sample. The NAAT result is reported as the Ct value. The smaller the Ct value obtained by the test, the higher the concentration of the marker in the sample, indicating a higher viral load of the subject [15]. According to the Ct value of the NAAT results, the samples are divided into strong positive, medium positive and weak positive groups. The positive and negative agreement rates between the antigen test and the nucleic acid test in different groups are calculated respectively. The stability and detection ability of the Ag-RDT results are confirmed by stratified statistical analysis.

## Main considerations for clinical research design

The design of the clinical trial for the evaluation of antigen detection reagents is directly related to the purpose of the study. As direct evidence for infection, sensitivity and specificity are the most concerned indicators in clinical performance of the tests. Identification of the applicable population in the intended use and applicable scenarios in which the antigen reagent can play a role in disease diagnosis is critical to the design of such clinical trials.

### Why should early infection cases be enrolled?

The main advantages of antigen detection are short detection time and convenient operation. For example, immunochromatography reagent only takes about 15–20 min to obtain results. However, the sensitivity of the antigen detection method is lower compared with NAATs. The advantage of the intended use of an antigen detection reagent is the rapid detection of infection, not monitoring of infection during treatment. The enrollment criteria of clinical trials should be formulated according to intended use population of the product and should simulate to the extent possible the clinical use scenario when the product is post market. Therefore, the positive population during the clinical trial of the Ag-RDTs should mainly be targeted at patients in the early stage of infection, and the clinical performance of the reagent in the intended use population should be evaluated. In clinical trials, the course of infection and onset of symptoms of study subjects are obtained from their recorded clinical history. Samples obtained within 7 days of the onset of symptoms are considered positive cases for early infection [16].

### Why do we need to use freshly collected samples?

It should be noted that in the instructions for Ag-RDTs, the requirements for sample collection, storage and sample processing reagents used in conjunction with them are different from those of NAATs. NAATs mostly use directly inactivated sample collection tubes, and the use of such samples for antigen detection may affect the detection results [17]. Therefore, residual samples routinely used in NAATs cannot be directly for antigen reagent clinical trials. The samples used in the clinical trials for antigen tests need to be collected, stored, processed and tested in accordance with the relevant requirements in the instructions, and nucleic acid testing should be performed simultaneously.

### Why should the antigen test results be compared with the nucleic acid test result in addition to the result of the diagnostic reference standard?

Many scholars have carried out clinical research on SARS-CoV-2 antigen detection reagents. The performance of antigen detection reagents obtained from different studies are quite different. It is difficult to verify whether these differences are due to differences in the performance of the reagents or due to gaps in clinical research design and included cases. Because of the differences in the design and enrolled study subjects, results from different clinical studies are not comparable. The sensitivity and specificity of products based on different groups of cases do not represent the overall performance of antigen reagents. When more weak positive cases are included, the clinical sensitivity will decrease significantly [18,19]. However, if NAATs are used to analyze the positive intervals of the same sample simultaneously, results from the antigen test can be presented better.

### Why do we need to carry out stratified statistics of strong, medium & weak positive samples?

As discussed previously, it is not difficult to find that if more strong positive samples (based on the sample’s own nucleic acid test results) are included, the positive detection rate and the clinical sensitivity of the antigen test will be higher [20–22]. The increased sensitivity, however, is likely due to selection bias of the enrolled subjects rather than a true reflection of the clinical performance of the antigen detection product. Stratified statistical analysis of positive samples can be performed to evaluate differences in clinical performance among different antigen products. For samples with different viral loads, a certain Ct value range requirement can be set, such as Ct ≤ 25 and Ct ≤ 30, to calculate the positive rate of antigen reagents. Through such stratified statistics, the performance of antigen reagents can be analyzed.

## Review practice

Clinical trials for three products have been completed based on the clinical trial requirements discussed previously, and these products were approved to the market by the NMPA at the time of revising the manuscript on 8 February 2022. The authors found that different products with different methodologies all had better sensitivity in strong positive samples. As the Ct value of the sample NAATs increases – that is, the positive strength decreases – the positive detection rate of antigen test reagents gradually decreases.

Taking the clinical data of one of products approved for marketing by the NMPA as an example, the clinical study enrolled a total of 502 cases. The test results showed that the positive agreement rate of the product with the nucleic acid test results was 60.8% (95% CI: 52.04–68.91%), and the negative agreement rate was 96.29% (95% CI: 93.86–97.78%); the overall agreement rate was 87.45% (95% CI: 84.26–90.07%). From the overall data, the positive detection rate of the Ag-RDTs does not seem to be very good. If stratified analysis is performed, however, detailed performance results of the product can be obtained. The positive agreement rate of this product is 86.90% (95% CI: 78.05–92.53%) for samples with a Ct value of the ORF1a/b gene of the nucleic acid test less than 30, whereas for samples with a Ct value of the ORF1a/b gene of the nucleic acid test less than 25, the positive agreement rate of this product is 98.18% (95% CI: 90.39–99.68%). The positive detection rate of the product steadily increases with the intensity of the positivity. The types of sample for clinical evaluation include oropharyngeal swabs and nasopharyngeal swabs. After preliminary evaluation, the authors confirmed that the clinical performance of the product can meet the emergency needs of the epidemic. On the contrary, if the detection result of the antigen test reagent for the strong positive samples in the clinical trial is not satisfactory, it can be inferred that the performance of the product does not meet the requirements for clinical use.

This analysis also clearly illustrates the clinical performance of Ag-RDTs. Compared with NAATs, the sensitivity of antigen detection is slightly inferior, which is due to methodological differences. The antigen detection method, however, has its own advantages. The operation is simple, which does not require a PCR laboratory. The detection time is short, and the result is available quickly. Based on the preceding circumstances, the NMPA defines the intended use of antigen testing as the rapid triage and preliminary testing of patients in the early stage of infection when the epidemic is concentrated, not as a basis for diagnosis and exclusion. It will help to quickly identify infected individuals and conduct isolation and monitoring in a timely manner. Based on the difference in sensitivity between antigen detection and NAATs, antigen detection cannot replace NAATs for diagnosis and monitoring of COVID-19.

## Limitations

The Key Points document and clinical trial requirements mentioned in this article are only for the premarket clinical research of SARS-CoV-2 Ag-RDTs, and they do not cover postmarket research and other research requirements. Because of the urgent need for SARS-CoV-2 testing reagents, premarket clinical studies can be limited in scope for initial analysis of the clinical performance of the product. Moreover, different countries also have differences in the intended use of detection reagents based on differences in their antiepidemic policies and detection capabilities. Special attention should be paid when using antigen reagents, as they may miss weak positive samples as screen methods due to their sensitivities and differences in viral load at different stages of the SARS-CoV-2 infection. For inspection or diagnostic purposes, this false negative result may result in a wide spread of the infection, Therefore, Ag-RDTs should not be used as the only diagnostic test method [23–26].

## Conclusion

Through meticulously designed clinical studies, it is possible to compensate for the differences in different clinical trials due to the differences in the included samples, and to analyze the clinical performance of different reagents horizontally. The clinical research results can reflect the overall clinical performance of the Ag-RDTs, thereby providing a more accurate reference for its clinical application.

Through clinical studies of multiple products, it is found that Ag-RDTs can obtain a better detection rate in the detection of strong positive samples, but the detection rate of weak positive samples will decrease. When describing the clinical performance of antigen detection reagents, it is not recommended to simply describe its sensitivity and specificity. Details of the study population and sample conditions on which the sensitivity and specificity are based should be provided. Different clinical studies cannot be directly compared if the population and sample conditions are unknown, nor can they reflect the performance differences among reagents.

The scope of clinical application of SARS-CoV-2 Ag-RDTs should be considered carefully based on their clinical performance.

## Future perspective

As a traditional method for the diagnosis of infectious diseases, antigen detection plays an important role in the diagnosis of many diseases. Even with the rapid development of NAATs in recent years, antigen detection still occupies an important position in clinical diagnosis because it is simple, rapid and direct. In the diagnosis of a serious infectious disease such as COVID-19, the use of NAATs is undoubtedly the more important method of detection and diagnosis, but other methods such as antigen detection and antibody detection can also play a role according to their own performance characteristics [27]. The sensitivity of antigen detection, however, still needs to be continuously improved. In the process of product development and verification and validation, correct evaluation of product performance is the key basis for the correct application of products to clinical diagnosis. When choosing products in clinical use, one should not consider sensitivity as the only indicator of performance. Clinical research results provided by the manufacturer, the local epidemic prevention policy and the local morbidity rate should be considered comprehensively [25,28].

For areas with high community disease transmission rates, the use of Ag-RDTs for rapid identification of infected individuals will help control the spread of the epidemic more quickly, and in remote and backward areas where there are no PCR instruments, antigen detection is a good option.

**Executive summary**

- Severe acute respiratory syndrome coronavirus 2 (SARS-CoV-2) antigen-detecting rapid diagnostic tests (Ag-RDTs) have many advantages, such as easy storage and transportation, simple operation, and low requirements for equipment and environment in the coronavirus disease 2019 (COVID-19) pandemic.
- The Center for Medical Device Evaluation of the National Medical Products Administration (NMPA) issued a document on ‘Key Points of Technical Review for Registration of SARS-CoV-2 Antigen/Antibody Detection Reagents.’
- Based on the Key Points document, this article focuses on the clinical research to explain the evaluation methods and requirements of clinical performance of SARS-CoV-2 antigen detection reagents, then analyzes the application scenarios and intended use of antigen detection reagents in the diagnosis of SARS-CoV-2 infections.

**Determination of clinical reference methods**

- The Key Points document stipulates the diagnosis and treatment guidance issued by the National Health Commission of China as the reference system.
- Analysis of the performance gap between antigen detection results and nucleic acid amplification tests (NAATs) results is important in understanding the role of antigen detection in diagnosis of COVID-19.

**Subject requirements**

- In clinical trials of Ag-RDTs, suspected cases of COVID-19 should be included. The initial stage of infection is patients with clinical symptoms within 1 week.

**Sample collection requirements**

- Ag-RDTs are expected to use throat swab and nasopharyngeal swab samples. Freshly collected samples should be used.

**Sample size**

- It is recommended that the size of the enrolled samples be estimated using an appropriate statistical model considering the preset values of clinical evaluation indicators.
- It should be avoided to include patients at a certain time point, such as 3–5 days after infection, and to avoid centralized inclusion of strong positive samples.

**Data integrity requirements**

- Clinical trial data should include detailed and complete clinical background of the subject, including the time of onset of clinical symptoms, time of visit, time when samples were taken, sample type, and time of the antigen and nucleic acid test as well as results.

**Statistical analysis**

- In general, 2 × 2 contingency tables are used to display the results and calculate clinical sensitivity and clinical specificity.
- According to the Ct value of the NAAT results, the samples are divided into strong positive, medium positive and weak positive groups.
- The positive and negative agreement rates between the antigen test and the nucleic acid test in different groups are calculated, respectively.

**Main considerations for clinical research design**

- The positive population during the clinical trial of the Ag-RDTs should mainly be targeted at patients in the early stage of infection, within 7 days of the onset of symptoms, and the clinical performance of the reagent in the intended use population should be evaluated.
- Residual samples routinely used in NAATs cannot be directly for antigen reagent clinical trials.
- The sensitivity and specificity of products based on different trials do not represent the real clinical performance of antigen reagents. However, if NAATs are used to analyze the positive intervals of the same sample simultaneously, results from the antigen test can be presented better.
- As the measurement of viral loads, a certain Ct value range requirement of NAATs result can be set, such as Ct ≤ 25 and Ct ≤ 30, to calculate the positive rate of antigen reagents. Through such stratified statistics, the performance of antigen reagents can be analyzed.

**Review practice**

- Clinical trials for three products have been completed based on the clinical trial requirements discussed previously, and these products were approved to the market by the NMPA at the time of revising the manuscript on 8 February 2022.
- Taking the clinical data of one of the products approved for marketing by the NMPA as an example, the clinical study enrolled a total of 502 cases.
- The types of sample for clinical evaluation include oropharyngeal swabs and nasopharyngeal swabs.
- After preliminary evaluation, the authors confirmed that the clinical performance of the product can meet the emergency needs of the epidemic.

**Limitations**

- The Key Points document and clinical trial requirements mentioned in this article are only for the premarket clinical research of SARS-CoV-2 Ag-RDTs, and they do not cover postmarket research and other research requirements.

**Conclusion**

- Through clinical studies of multiple products, it can be found that Ag-RDTs can obtain a better detection rate in the detection of strong positive samples, but the detection rate of weak positive samples will decrease.
- The scope of clinical application of SARS-CoV-2 Ag-RDTs should be considered carefully based on their clinical performance.

**Future perspective**

- As a traditional method for the diagnosis of infectious diseases, antigen detection plays an important role in the diagnosis of many diseases.
- The sensitivity of antigen detection, however, still needs to be continuously improved.
- Clinical research results provided by the manufacturer, the local epidemic prevention policy and the local morbidity rate should be considered comprehensively.
